# Address allocation for MANET merge and partition using cluster based routing

**DOI:** 10.1186/2193-1801-3-605

**Published:** 2014-10-16

**Authors:** Sugandha Singh, Navin Rajpal, Ashok Sharma

**Affiliations:** University School of Information and Communication Technology, GGSIPU, Delhi, India; YMCA University, Faridabad, Haryana India

**Keywords:** MANET, Network merging, Network partitioning, Clustering

## Abstract

Network merges and partitions occur quite often in MANET wherein address auto-configuration is a critical requirement. There are various approaches for address auto-configuration in MANETs which allocate address to the nodes in a dynamic and distributed manner in which HOST ID and MANET ID are assigned on the basis of their Base value. MANET merges and partitions employing Cluster Based Routing Protocol require a node to be assigned as the Cluster Head (CH). This paper presents the Election Algorithm which assigns a node as the Cluster Head on the basis of its weight. Through simulation using the NS-2, it has been shown that the Election Algorithm improves the packet delivery ratio (PDR) significantly and decreases the packet delay to a great extent in comparison to the existing AODV protocol.

## 1 Introduction

Mobile Ad hoc Network (MANET) is dependent on the wireless technology and thus relies on the wireless transmitting devices. Wireless channels are used for communication among these devices without any assistance from fixed or structured infrastructure. The network consists of only nodes, and thus central management of the network is not necessary in guiding the nodes on how to communicate. The nodes act as routers and cooperate among themselves to facilitate communication. There are various applications in which the ad hoc networks can be used. These include the military operations, disaster situations etc. Additionally, MANET’s can be used in the local mobile connectivity, Educational applications and Wireless Sensor Networks among others.

Network interfaces for the deployment of MANET’s in various applications require the support of the network routing protocol. The primary requirement of any network is the delivery of correct message within time between the nodes and a routing protocol ensures the establishment of the route between them. Further, the functionalities of the network are completely dependent on the IP addresses of the nodes. It is also critical to ascertain if some nodes within the network have a similar IP address as it can inhibit the smooth functioning of the network.

There is a limitation of manual allocation of unique identifier due to random movement of nodes which leads to frequent changes in topology. This issue could be overcome through the address auto-configuration approach. (Indrasinghe et al. 2007).

Address Auto-configuration Approaches – These approaches automatically assigns IP addresses through auto configuration. Any address auto-configuration mechanism should meet the following requirements:

- Topology change- In MANET’s nodes are mobile and could join and leave the network at any moment without notification. While designing an auto configuration mechanism this dynamism of network topology should be considered.
- Network Partitioning and Merging- The movement of nodes of an ad hoc network could divide the network in two or more disconnected networks, which is called network partitioning. These partitions or other mobile networks could remerge later. The auto-configuration protocol should be able to deal with these situations and the resulting address conflicts or address leaks (Webhi 2005).

IP address auto-configuration could be categorised into stateless (Perkins et al. 2001), stateful (ul Huq et al. 2010; Mohsin and Prakash 2002) and hybrid approaches (Indrasinghe et al. 2008).

This paper considers address allocation through auto-configuration. It assigns IP addresses automatically and also gives the characteristic comparison of the stateless, stateful and hybrid protocols. For handling the frequent partitioning and merging of network due to high mobility of the nodes, a hybrid routing technique named Election Algorithm for selecting Cluster Head (CH) is proposed. It is also shown that clustering structure decreases routing control overhead, improve the network scalability, mobile coverage reliability and cluster head reliability. Results of our simulation show that there has been significant increase in PDR and considerable decrease in packet delay. Paper is organized as follows:

Section 2 gives the characteristic comparison on various address auto-configuration protocols. Section 3 describes the various addressing architectures for cluster based MANET merge and partition. Section 4 proposes the Election Algorithm for cluster head selection. Section 5 explains the proposed states transitions of nodes in NORMAL state address configuration through the flow charts. Section 6 shows the simulation results and also explains the reason for considering AODV for comparison. Section 7 concludes the work and briefs about the future work.

## 2 Characteristics comparison of address auto-configuration protocols

The existing approaches are based on the following factors which are compared and shown in Table 1.

- Address Evenness- An effective address distribution means better utilization of address space as the available address space is limited. An even distribution indicates low address duplication probability and better utilization of address space. Hence, this metric is the measurement of protocol effectiveness on address distribution. For all existing auto-configuration approaches, address evenness is achieved by design; the only exception is for the Buddy protocol. In Table  1, protocol that achieves evenness by design is indicated as "yes" and a protocol that shows unevenness or achieves evenness by additional measures is indicated as "no".
- Routing protocol dependency – If any approach is dependent on any specific routing protocol then it is considered with better design and performance, however, independency leads to flexibility.
- Distributed operation- In MANET’s distributed operation is always preferred. Accordingly, a certain level of centralization can be tolerated but at the same time the potential effects of such centralization should also be considered.
- Address uniqueness- It is the factor which if not in working, may adversely affect the security and can cause network perturbation.
- Address stability- Unnecessary address changes leads to instability in network and overhead for assigning new addresses. Address stability would lead to users’ satisfaction and prevent corruption of active communications.

**Table 1:** **Characteristics comparison between existing protocols**

	Address evenness	Routing dependency	Distributed operation	Address uniqueness	Address stability	Approach
Agent based addressing (Günes and Reibel 2002)	Yes	No	Centralized	Guaranteed	Low stability	Stateful
MANET Conf. (Nesargi and Prakash 2002)	Yes	No	Distributed	Guaranteed	High stability	Stateful
Prophet (Zhou et al. 2003)	Yes	No	Distributed	Not guaranteed	Not specified	Stateful
Buddy protocol (Mohsin and Prakash 2002)	No	No	Distributed	Guaranteed	High stability	Stateful
Strong duplicate address detection (Perkins et al. 2001)	Yes	No	Distributed	Not guaranteed	Not specified	Stateless
Weak duplicate address detection (Vaidya 2002)	Yes	Yes	Distributed	Guaranteed with high Probability	High stability	Stateless
Passive duplicate address detection (Weniger 2003)	Yes	Integrated within the routing protocol	Distributed	Guaranteed with high Probability	High stability	Stateless
Adhoc IP address autoconf. (Jeong et al. 2005)	Yes	Yes	Distributed	Guaranteed with high Probability	High stability	Stateless
Hybrid centralized query based auto-configuration (Sun and Belding-Royer 2003)	Yes	No	Semi centralized	Guaranteed	High stability	Hybrid

On the above mentioned parameters the protocols are compared in Table 1. On the basis of this comparison and considering the parameters addressing architecture is explained in section 3.

## 3 Addressing architecture for cluster based MANET merge and partition

The purpose of address is to provide identification. It does not provide any information about the topological location of a node within the network. The most fundamental property of a network is the way it assigns address to the nodes so as to facilitate communication among the nodes. As the movement of the nodes in MANET is independent of any other node in the network, so, the node also acts as an independent router.

Notably, one node can have numerous physical network interfaces. Generally, in an IP network, each node will have a different IP address. However, the need is to provide unique identifier to each node in MANET by which each node will be known to others within the network. Therefore, it is required to assign unique IP address to each of the participating node from the pool. As per the mobile IP terminology, this address is referred as a node’s home address. To distinguish the nodes between the multiple network interfaces, some other notation in the form of locally assigned interface index is also required in addition to the IP address.

### a) Node address assignment

It should be noted that, since hosts from various MANETs can be in contact with one another, they have to indicate that they come from different MANETs mentioning the address used. Otherwise, there is the use of the tunnel in transferring data between the nodes belonging to various MANETs. Nonetheless, there is a possibility of two distinct MANETs having a similar MANET ID. In case there are incidences where two MANETs with a similar ID come into contact, there should be a distinct mechanism to distinguish them. Hence MANETs should also have a identification, distinct from their IP addresses. Here it is proposed that address space should be structured into MANET ID and Host ID.

To address the host within the MANET the private addresses of IPv4 are used. (Indrasinghe et al. 2008). The blocks used are:


For elucidation, block of 10/8 prefix is used. This block avails 24 bits that can be addressed. If a third of the bits are used for the MANET ID, there is 1/256 chance that two MANETs will have a similar ID. In this case, 16 bits are left for host ID. This means that 64 K hosts are available for the MANET. In this mechanism, there is a distributed algorithm in the allocation of the remainder of the address space to the hosts. In this case, it will be 16 bit address space (Indrasinghe et al. 2007). According to this mechanism, all mobile hosts within the network can generate various addresses. In addition, the address ranges are fragmented for two hosts in the MANET. Similarly, each of the hosts produces unique numbers. Also, the number of unique addresses produced is dependent on the selected *Base Value*. The numbering of the hosts depends on the sequence in which the host arrives in the network. When the host leaves the network number is re-used (Indrasinghe et al. 2008). The following numbers are generated from any given host, whose number is *n.*1

Here

Base Value = In this illustration it is taken as 3 else it could be 2,3,4….


The values obtained in Eq. (1) are then added to the first address in the host range to generate a new address.

**Figure 1:**
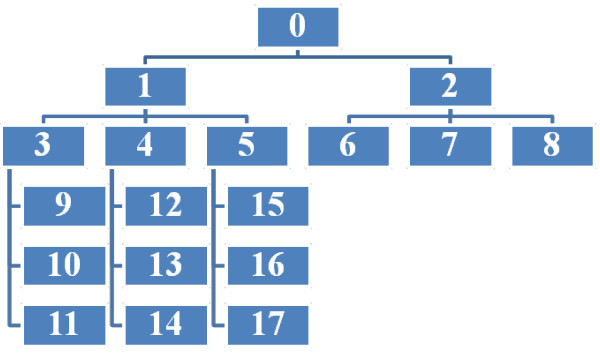
**Address allocation tree with the base value of 3.**

Address generation is shown in a tree structure in Figure 1, for the base value 3. The sequence in which addresses are generated is represented in Table 2. The case of MANET merge and partition is discussed in next paragraph where the situation of graceful portioning is taken into consideration. As we increase the base value, the number of nodes in the single network increases indicating the wide tree structure with more leaves, therefore, the parent host has to deal with more address re-allocations.

**Table 2:** Node ID assignment for base value 3

Host ID	i value	Generated value
0	0	0
	1	1
	2	2
1	0	3
	1	4
	2	5
2	0	6
	1	7
	2	8
3	0	9
	1	10
	2	11
4	0	12
	1	13
	2	14
5	0	15
	1	16
	2	17

### b) MANET partitioning

Network partition involves the movement of nodes for leaving the network. Partitioning could be abrupt or graceful. Abrupt partitioning has not been dealt in this paper. Partitioned networks need to update reachable host tables in the new network. Therefore, for correct and efficient network communication, the new network hosts are to perform addresses clean up through entire parent network as per the new topology changes. MANET when splits into partitions then they are called clusters and the cluster heads of these partitions are to be elected which initially will be the same for both but now is part of one only (possibly). These clusters may merge and leads to development of a bigger cluster/MANET. Partitioned networks need to act as an independent network so one partitioned network requires to alter its cluster head (Lee et al. 2007). In the proposed addressing scheme, structured MANET ID is allocated with 8 bits. The possibilities of having unique MANET ID are 256 in case the MANET ID is of 8 bits.

On arrival, host requires a new address which is done by using broadcast method. It assigns addresses by detecting the missing host address in tree structure after receiving the acknowledgement of each existing host. Thus, the parent will also be informed of the missing host.

### c) MANET merging

MANET merge is a frequent activity. It may involve the networks which are independent or had been partitioned earlier. Prior to merge, each network has independent configured addresses. When the networks merge, two or more networks might have the same addresses leading to address conflict. Hence, for efficient and correct communication, it is necessary to resolve this situation.

The importance of an alternative approach to redress this situation has also been identified in this research. When two distant nodes named N1 and N2 of different networks come within communication range of each other, they exchange their MANET identities. As the received and sent MANET identities are different, the larger MANET adopts the smaller one and starts allocating the new addresses for the newly created MANET. The protocol needs to be developed for assigning IP addresses without disrupting on-going communications. To resolve this situation, following mechanism has been developed.

When each MANET is named with, say, eight random characters then the probability sets close to 1 for the two merging MANET’s for not to have the same ID. The purpose of MANET ID is to differentiate the nodes belonging to different networks.

Now when two Clusters merge, which were earlier partitioned to different clusters must have the same MANET ID but have independent cluster heads (CH). When merging and partitioning occurs, CH has to be elected. Here the election algorithm for selecting the node as the cluster head (CH) is proposed and assign the cluster head ID as ID_ch_ to that node to inform all the neighbour nodes about their cluster head.

## 4 Cluster head selection

**Figure 2:**
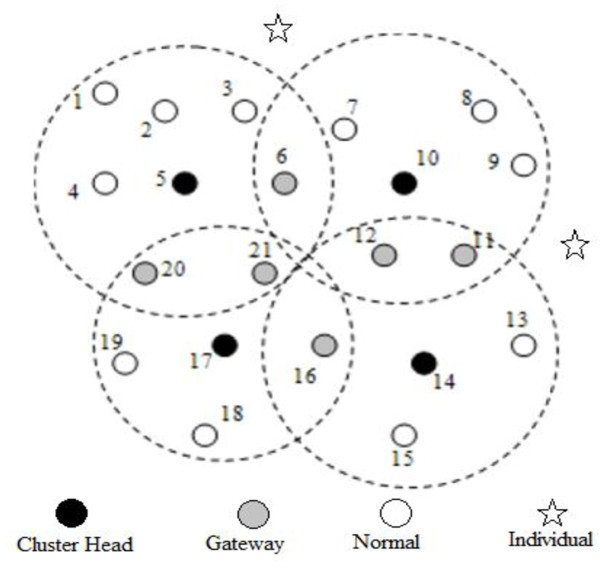
**Cluster formation.**

The process of dividing the network into interconnected sub-networks (clusters) is called clustering. In the proposed scheme, each cluster selects the cluster head (CH) which acts as coordinator for that cluster (Wang and Hung 2013). Prior works on clustering focus on maintaining a stable link between the clusterhead and its members. They measure the velocity of the clusterhead (Chatterjee et al. 2000, 2002). In the proposed algorithm, nodes can be in any of the following states:

- INDIVIDUAL: Node is INDIVIDUAL when it is not in proximity of any MANET and is in need of any of the cluster.
- NORMAL: Node is said to be in NORMAL state when it is in proximity of one cluster head only and is part of one MANET/Cluster.
- CLUSTERHEAD (CH): Node is CH when it is being selected through election algorithm as the head of a particular cluster.
- GATEWAY: Node is GATEWAY when it is in proximity of more than one cluster and comes in contact with more than one cluster head.
  The nodes mentioned above are shown in Figure  2 with their possible positions, showing one type of cluster formation. Initially all nodes are in this state. Each node maintains the NEIGHBOR table wherein the information about the other neighbor nodes, within their range, is stored. CHs have another table CHNEIGHBOR, wherein the information about the other neighbor CHs is stored. The primary step in clustering is the CH election.

### Election algorithm

Algorithm 1 describes the overall mechanism for selecting cluster head. This algorithm allocates the weight to every node. For a node to become CH, its overall weight (W) is to be measured. The node which sends the message to neighbour earlier than others and is in the higher group would be chosen as CH. Periodically LIVE message is broadcasted by every node for announcement of its existence. Weight is calculated as in equ. 2.
2

N: It is the value which indicates the number of neighbors of that node.

R: It is the remaining battery lifetime of node.

T: It is the cumulative time, which indicates the life of that node in the previous cluster.

P: Transmission Power.


If the transmission power level of the received node is greater than the previous received node, then the node would be considered in the pool of N. This ensures the cluster that node is moving closer. The probability of a node to become CH would be higher with the larger value of N. The value of T directly indicates the node stability and average speed of node during specified time. Node with maximum P will be elected as CH as it can cover the largest range. The probability of a node to be selected as CH is calculated in equ 3:
3

P_vi_ : It is the probability of each node to be selected as clusterhead.

α : It is relative importance of visibility measure.

β : It controls the number of times same path is used.

W_vi_ : It is the weight of the visible node i.

Neighbor table is maintained by each node depending upon the information it receives from its neighbours. Time T_e_ is defined for selecting CH. If any CH sends the LIVE message to any INDIVIDUAL node within T_e_ then it sets ID_CH_ with its own ID and replaces the node state from INDIVIDUAL to NORMAL. A node may also elect itself as CH and sets its state to CLUSTERHEAD in case:

- If a node does not receive LIVE message from any CH and its neighbour, where the node with higher parameter (W) than itself is searched within time T_e_.
- Else it continues to send LIVE message until 2T_e_ time and then declare itself as CH.

In this algorithm, when the two meeting Clusterheads nodes A and B come close within one hop then the cluster head change event is initiated. In this event, the node which gets the LIVE message first checks its neighbour table. If all member nodes are GATEWAYs, it changes the state to a NORMAL and sets ID_ch_ with address of B else it checks its W parameters. The node with lower W parameter is set as GATEWAY and the other one update itself as ID_CH_. If A finds that it has higher W parameters, it sends a unicast COVERLAP message to B. Then B terminates its clusterhead role and changes the state to GATEWAY and sets ID_ch_ with the address of A.

When a node S attempts to deliver data to another node D, which is not in its routing table then it first check its neighbor table, if node is found then it send the data to D and add this route into routing table. Else it initiates a path discovery process to locate D.

## 5 Node working in NORMAL mode

**Figure 3:**
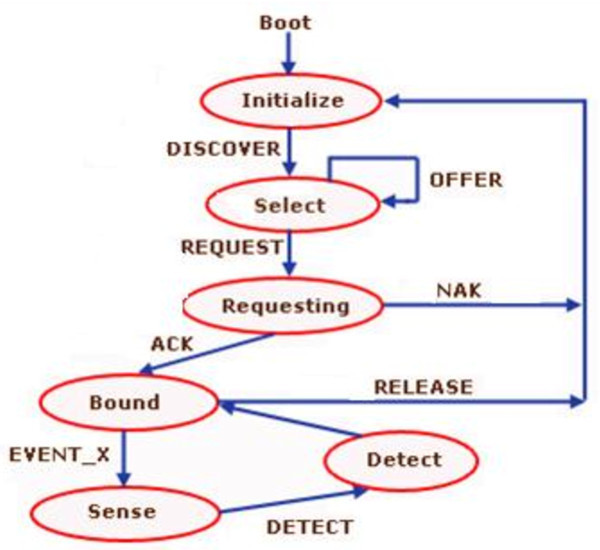
**Transition diagram for new node.**

Network is initiated when root is declared. Figure 3 represents the process for the new node (Kumar and Singla 2009).

- A join request message is broadcasted to all the neighbouring nodes of the new node *(N*_*i*_*),* which wants to join the network by activating the message, DISCOVER. Here it enters into the "select" state, where it waits for certain amount of time. Two outcomes are possible:
  i. If the reply of this request is not received before expiry of this timer than the request is sent again. If a node doesn’t receive a response even after ‘*n*’ number of tries then node considers that it is the first node and announces itself as the root of MANET.
  ii. One or more offers can be received by the new node and thereafter it goes to the select stage.
- In the select stage, a node with the smallest address and maximum weight is selected among the offered and sent with the REQUEST.
- If node receives an acknowledgement (ACK) before the expiry of requested timer then it goes to bound stage, where it configures itself and starts various timers for further MANET communication.
- Else if it receives negative acknowledgment (NAK) or timer expires then it restart the initialize process.
- If MANET merge and partitioning (EVENT_X) is detected then node activates a sense process to determine the merge or partition and sends a DETECT message to the bound node. To ensure detection of partitioning and merging, the oldest node in the MANET should start the DETECT process. There are two major cases in MANET partitioning:
  i. When Ni wants to leave the network than it send the RELEASE message to the parent node. In case parent node is in the network then it responds back to the node Ni and keeps the address of Ni for recycle/reuse. But if parent has already left the network then the root node deals with the RELEASE request and keeps the address for recycle.
  ii. If the node has left the network abruptly than the address release is detected during the partition detection process.

**Figure 4:**
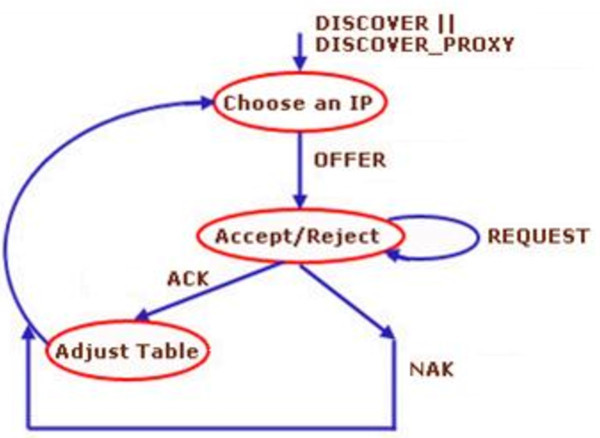
**Transition diagram for root and proxy node.**

The state diagram for root and proxy nodes is represented in Figure  4.

- If a node detect the DISCOVER message then it chooses an IP and send an OFFER message to the new node and wait for REQUEST message.
- On REQUEST from the new node, it decides to accept or reject the request and accordingly sends acknowledgment (ACK) or negative acknowledgment (NAK). The assignment of the address depends upon the availability of address in the pool. After assigning the address accordingly the table is adjusted.

## 6 Simulation

The proposed routing protocol is evaluated and compared with AODV routing protocol by simulation. The proposed algorithm is implemented on the NS-2 simulator in terms of packet delivery ratio (PDR) and the average end to end delay.

For Comparing Election Algorithm, AODV is used for the reason that AODV is another variant of classical distance vector routing algorithm, a confluence of both DSDV and DSR. It borrows the basic on demand mechanism of route discovery and route maintenance from DSR, plus the use of hop by hop routing, sequence numbers and periodic beacons from DSDV. AODV is used for comparison for being having the following characteristics:

i) In AODV whenever a route is available from source to destination, it does not add any overhead to the packets. However, route discovery process is only initiated when routes are not used and/or they expired and consequently discarded.
ii) It also has the ability to provide unicast, multicast and broadcast communication as it uses the broadcast route discovery algorithm and then the unicast route reply message.

Election Algorithm is compared on the basis of packet delivery ratio (PDR), which is the ratio of the number of packets received by the destination to the number of packets originated by the source. It specifies the packet loss rate, which limits the maximum throughput of the network. The higher the delivery ratio, better the routing protocol.

The input parameters are as listed in Table 3. The number of mobile nodes is set to 50 to 300 nodes. These nodes are spread randomly in a 500 m × 500 m area network.

**Figure 5:**
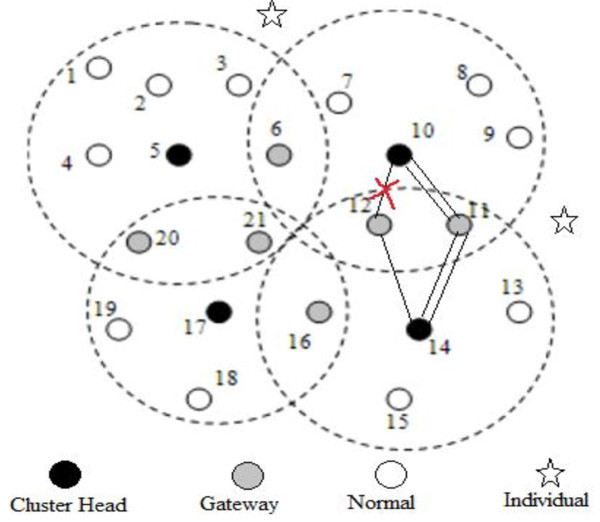
**Mobile adhoc network with arbitrarily chosen range of 200.**

Algorithm 1 was iterated equal to the number of nodes for an optimal solution. Once a node is selected as the cluster head, all the nodes which are in vicinity of CH and one hop away will be covered and thus becomes part of the cluster. The model of the ad hoc network developed for 200 nodes is shown in Figure 5. The nodes are shown as circles with an identifier associated with each node.

**Table 3:** **Simulation parameters**

Parameter	Value
Nodes number range	50–300
Network size	500 m × 500 m
Nodes average speed	5–20 Km/h
Transmission range	30–300 m
α	9
β	1

Table 3 shows the EA parameters used. Node starts from a random location to a random destination point with its specific speed. As the node reaches its destination after pause time another destination is targeted.

**Figure 6:**
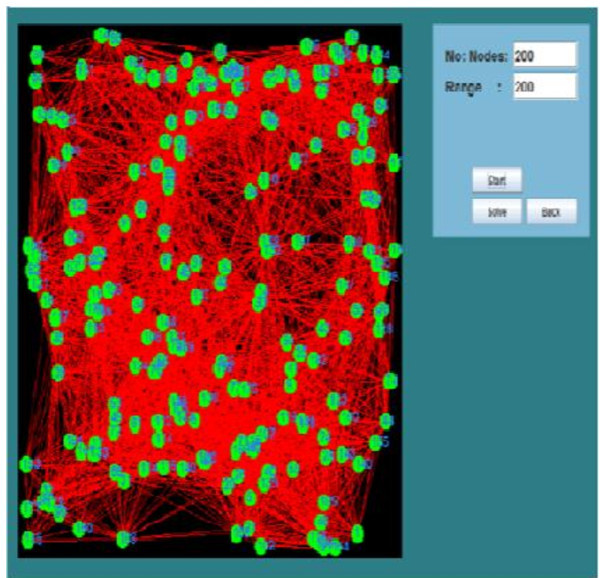
**Adhoc network of 200 nodes with 7 are clusterheads.**

Further, the formation of cluster with 200 nodes is shown here in Figure 6. The nodes blue in color are the Clusterheads.

**Figure 7:**
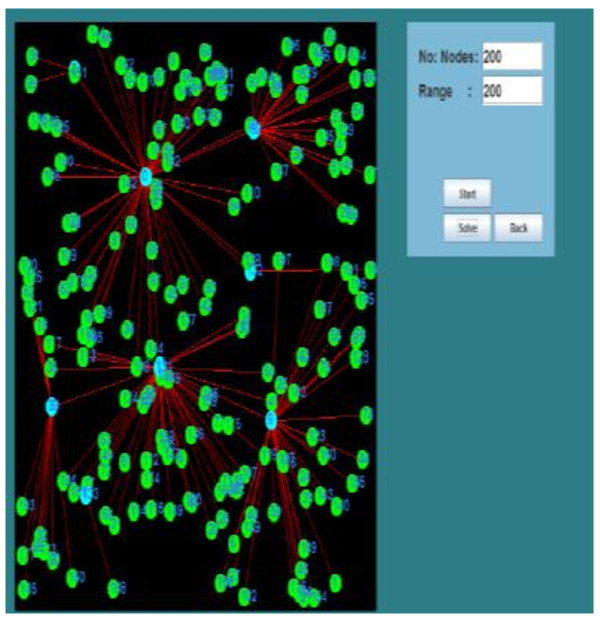
**PDR vs. number of nodes.**

Generally, the PDR decreases with the increase in number of nodes. Figure 7 compares the PDR of CRP with AODV. It can be seen that the PDR of CRP increases significantly in comparison to the AODV protocol and the proposed Election Algorithm can scale up to a larger network.

**Figure 8:**
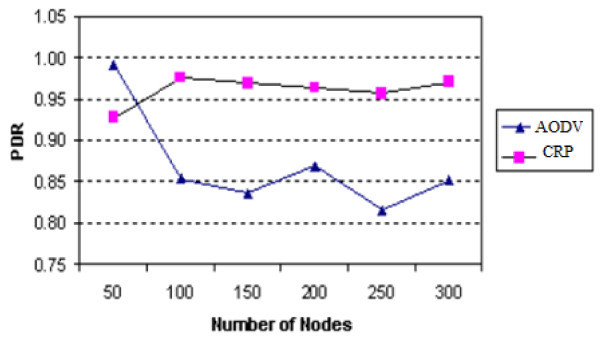
**PDR vs. node speed (50 nodes).**

Figure 8 shows the comparison of PDR of CRP and AODV at different node speeds. Probability of link failure increases as the node speed increases and therefore, the number of packet drops increases. However, the delivery ratio of CRP decreases slowly, in comparison to the corresponding drop in AODV. Hence the proposed method performs better.

**Figure 9:**
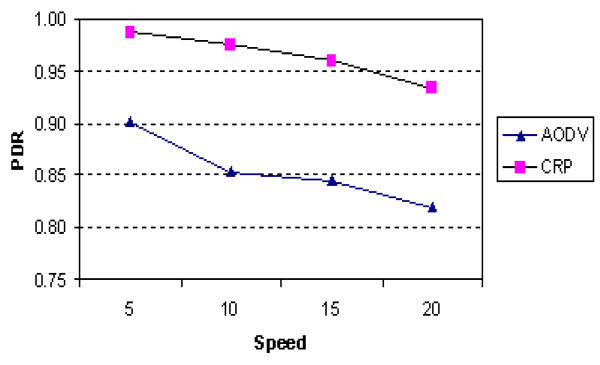
**Average end-to-end delay vs. number of nodes.**

There is increase in the average end-to-end delay with the increasing number of nodes because of appearance of more connections and congestions. Also, it could be inferred that the average end-to-end delay for proposed approach is better than the AODV protocol (Figure 9).

**Figure 10:**
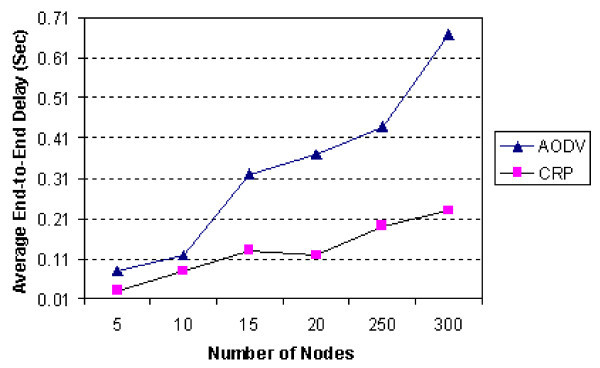
**PDR vs. pause time (50 nodes).**

Figure 10 shows the comparison of pause time and PDR. The PDR decreases due to the link breaks with increase in the node mobility. In this scenario also, the CRP performs better as compared to AODV.

**Figure 11:**
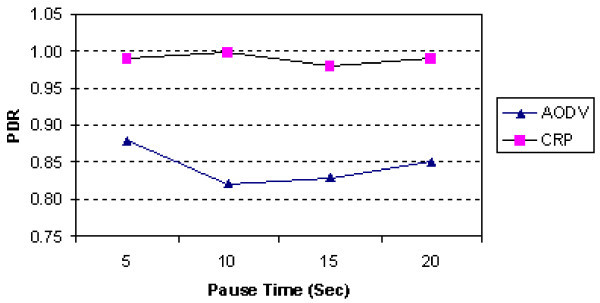
**Average end-to-end delay vs. pause time.**

Figure 11 compares the average end-to-end delay in various pause times. It is evident that the average end-to-end delay for both CRP and AODV routing increases with the decrease in the pause time due to frequent changes in the network topology.

## 7 Conclusion and future work

This paper proposes a new distributed algorithm (Election Algorithm for CRP) and a mechanism for assignment of unique address to new nodes joining a MANET. The working of the proposed model is shown through the flow chart in Figures 3 and 4 considering the merges and partitions of MANETs. The mechanism has used the existing address space for allocation of reusable addresses. The mechanism is capable in re-allocating root address even if the root host is unavailable. Also, a solution for MANET merges specifically in the case when MANET ID which is a part of the IP address is same for the merging MANET. Assigning a unique identifier for each MANET, distinguish its own nodes among hosts of other networks, thus could resolve the issue of IP address duplication in case of MANETs merger. In the previous methods if a node fails within a route or become far from its neighboring nodes, it causes the route to fail and leads to the recreation of another path. But in the proposed method, since the route is expressed by CH, in case a node fails in a route, the CH of that node can use another node to forward a packet to the next existing node in the route (Figure 5). In this method, when a cluster fails or corrupts only then the need of recreating of the path arises, which is in regard of the attempt to create more stable clusters. When a CH node detects a link break for the next hop CH of active route, it sends a route error packet back to all precursors. When a CH node receives a route error packet from a neighbor CH for one or more active route, it forward the packet to precursors stored in its route table. When a source node receives a route error packet, it initiates a new route discovery if the route is still needed.

Proposed Election Algorithm for MANET provide more access to network services and speed up the creation of clusters, due to consideration of weight nodes. When two clusters are located within the same range, then one of them will change its state to the state of GATEWAY. If in an existing route one node fails then instead of recreating the route CH uses another node to forward the packet. Through simulation it has been proved that PDR and performance of EA is higher over traditional protocols.

### Future work

Future work will include the evaluation on the basis of efficiency and performance at time of network partitioning and merging in address tree updating for different base values. A protocol is required for the clusters with same MANET ID to take the unique new IP address. This protocol is an issue for future endeavour in this research.
